# Comparative Performance of the MGISEQ-2000 and Illumina X-Ten Sequencing Platforms for Paleogenomics

**DOI:** 10.3389/fgene.2021.745508

**Published:** 2021-10-04

**Authors:** Kongyang Zhu, Panxin Du, Jianxue Xiong, Xiaoying Ren, Chang Sun, Yichen Tao, Yi Ding, Yiran Xu, Hailiang Meng, Chuan-Chao Wang, Shao-Qing Wen

**Affiliations:** ^1^State Key Laboratory of Cellular Stress Biology, School of Life Sciences, State Key Laboratory of Marine Environmental Science, Department of Anthropology and Ethnology, Institute of Anthropology, School of Sociology and Anthropology, Xiamen University, Xiamen, China; ^2^MOE Key Laboratory of Contemporary Anthropology, Department of Anthropology and Human Genetics, School of Life Sciences, Fudan University, Shanghai, China; ^3^Institute of Archaeological Science, Fudan University, Shanghai, China

**Keywords:** MGISEQ-2000, BGI tech, ancient DNA, paleogenomics, population genetics

## Abstract

The MGISEQ-2000 sequencer is widely used in various omics studies, but the performance of this platform for paleogenomics has not been evaluated. We here compare the performance of MGISEQ-2000 with the Illumina X-Ten on ancient human DNA using four samples from 1750BCE to 60CE. We found there were only slight differences between the two platforms in most parameters (duplication rate, sequencing bias, θ, δS, and λ). MGISEQ-2000 performed well on endogenous rate and library complexity although X-Ten had a higher average base quality and lower error rate. Our results suggest that MGISEQ-2000 and X-Ten have comparable performance, and MGISEQ-2000 can be an alternative platform for paleogenomics sequencing.

## Introduction

The last two decades witnessed a rapid development of genomics due to the emergence of next-generation sequencing (NGS) technology. Various NGS platforms based on different strategies have been developed, among which sequencing by synthesis-based Illumina’s NGS platforms has become the most widely used sequencing ones due to their high throughput and lower error rate. Although the cost of the Illumina-based platform is decreasing dramatically due to the development and refinement of NGS techniques, the low endogenous rate of ancient DNA (aDNA) is still limiting the paleogenomics studies.

In 2016, the Beijing Genomics Institution (BGI) launched its own NGS platform designated as BGISEQ-500 (Goodwin et al., 2016). The technology underlying the BGI platform combines DNA nanoball (DNB) with polymerase-based stepwise sequencing (Drmanac et al., 2010; Porreca, 2010). Then, BGI launched subsequent platforms, including BGISEQ-50, MGISEQ-200, MGISEQ-2000, and MGISEQ-T7. Among them, the MGISEQ-2000 platform was evaluated to be comparative in performance to Illumina NGS platforms in various studies, including whole-genome (Korostin et al., 2020; Jeon et al., 2021), whole-exome (Chen et al., 2019), single-cell transcriptome (Senabouth et al., 2020), and RNA sequencing (Jeon et al., 2019). MGISEQ-2000 has several features that may be valuable to the aDNA field. First, this platform has flexible read-length choices, such as SE50 (single-end), SE100, PE50 (paired-end), and PE100, which covers the peak size of the distributions of sequences reported from aDNA (Green et al., 2010; Allentoft et al., 2012; Orlando et al., 2013). Second, this instrument has a high throughput. With two flow cells, it can produce 720–800G base data within 48h by PE100 mode. Third, it has a lower data-producing cost: in general, about $10.8/G base data in the Illumina-based platform, and no more than $6.17/G in BGI in the sequencing market of China. At last, the similar laboratory workflow between the two platforms makes the procedures easily modified for aDNA. However, a comprehensive evaluation of the performance of MGISEQ-2000 in paleogenomics has not been reported.

To explore whether MGISEQ-2000 is a potential platform for paleogenomics studies, we analyzed whether there are significant differences between four samples sequenced by MGISEQ-2000 and X-Ten. We compared some key parameters that are crucial for paleogenomics studies and also directly compared the differences of samples from two platforms on population genetic structure. Our results suggest that MGISEQ-2000 from BGI Tech has a comparative performance with Illumina’s X-ten on several key parameters, which makes MGISEQ-2000 an alternative platform for generating aDNA data.

## Materials and Methods

### Archaeological Context and Skeletal Materials

We selected samples from two sites, named Mogou and Heishuiguo, from Gansu Province in northwestern China. The Mogou site is located in Lintan County, Gannan Tibetan Autonomous Prefecture (Dittmar et al., 2021). The cultural context of the Mogou site mainly belonged to Qijia and Siwa. The cemetery is located on a terrace above the southwest bank of the Tao River, and it covers more than 30 hectares. Radiocarbon dates indicate that the site was in use between 1750 and 1,100BCE. We sequenced one sample from the Mogou site in this study (Table 1).

**Table 1:** Samples from which aDNA was extracted. EA1102, EA1104, and EA1107 are three samples from different individuals.

Sample ID	Skeletal Element	Species	Locality	Age
EA1102	Petrous	Human	Ganzhou District, Gansu	100BCE to 60CE
EA1104	Teeth	Human	Ganzhou District, Gansu	100BCE to 60CE
EA1107	Petrous	Human	Ganzhou District, Gansu	100BCE to 60CE
F90914	Petrous	Human	Lintan County, Gansu	1750 to 1,100BCE

The Heishuiguo site is located in Ganzhou District, Zhangye City. This site was divided into six phases (G et al., 2019), spanning from the middle Western Han Dynasty to the Western Jin Dynasty (around 140BCE to 300CE). We sequenced three samples from the Heishuiguo site in this study, dated from the late Western Han Dynasty to the early Eastern Han Dynasty (around 100BCE to 60CE) based on the shape of the tomb and combination of burial articles.

### Laboratory Procedures

#### DNA Extraction

We extracted DNA from four samples in a dedicated aDNA facility at Fudan University, according to established precautions for working with ancient human DNA (Paabo, 1989; Knapp et al., 2012; Sun et al., 2021). For contamination monitoring, we included extraction negative controls (with which no sample powder was used) and library negative controls (with which the extract was supplemented by water) in every batch of samples processed and carried them through the entire wet laboratory processing. Before sampling, all samples were irradiated with UV light for 30min from all sides and wiped with 5% bleach. Then, teeth were sandblasted to remove the outer surface and ground to fine powder with the mixer mill (Retsch, Germany). We cut the dense part of petrous bones around the cochlea by first removing the outer part and then grinding the clean inner part into fine powder. We used 100mg of bone powder to extract DNA. The prelysis step included the addition of 1ml extraction buffer, containing 0.5M EDTA, 0.25mg/ml Proteinase K (Merck, Germany), pH 8.0, followed by 1h rotation at 37°C. After centrifugation, the supernatant was discarded, and 2.5ml extraction buffer was added followed by overnight rotation at 37°C. We mixed 20μl magnetic beads (Enlighten Biotech, China) with 12.5ml binding buffer containing 5M GuHCl, 40% Isopropanol, 25mM sodium acetate, 0.05% Tween-20 (Merck, Germany), pH 5.2. Then, we transferred the supernatant (~2.5ml) to a binding buffer/bead mixture followed by a robotic extraction (Enlighten Biotech, China) procedure. Finally, the DNA was eluted with 50μl TET buffer (QIAGEN, Germany).

#### Library Construction

We prepared double-stranded libraries following Meyer’s protocols (Meyer and Kircher, 2010; Bennett et al., 2014; Wales et al., 2015) but with minor corrections. Libraries were amplified with indexing primers in two parallel polymerase chain reactions (PCR) using Q5 High-Fidelity DNA Polymerase (NEB). Indexed products from the same library were pooled and purified using Agencourt AMPure XP beads (Beckman Coulter, Germany) and eluted in 20μl TET buffer. We qualified the clean-up libraries by Qubit 2.0 (Thermo Fisher, United States). We then sequenced a half volume of the libraries (~10μl) on an Illumina HiSeq X-Ten instrument at the Annoroad Company, China, in the 150-bp paired-end sequencing design. In the meantime, we converted the rest of the libraries (~10μl) into circular single-strand libraries adapted to the MGISEQ-2000 instrument, using the MGI Easy Universal Library Conversion Kit (App-A, Cat. No.: 1000004155). We then made DNBs and sequenced the libraries by the MGISEQ-2000RS High-throughput (Rapid) Sequencing Kit (App-A, PE100, Cat. No.: 1000005662).

### Data Analyses

#### Mapping and Subsampling

The processing of raw data followed the widely used PALEOMIX pipeline published in Nature Protocols (Schubert et al., 2014). The sequencing quality of raw data was first assessed using FastQC (Andrews, 2010). Raw reads were then trimmed using AdapterRemoval (v. 2.3.1; Schubert et al., 2016), with which consecutive stretches of the low-quality bases, Ns, and adapter sequences were trimmed from 5′ and 3′ termini. Raw reads from paired ends were merged, and only those overlapped by at least 11bp were retained (Wang et al., 2021). Besides this, reads that were shorter than 25bp were removed. The trimmed reads were then mapped to the human reference genome (hs37d5; GRCh37 with decoy sequences) using the backtrack algorithm implemented in Burrows-Wheeler Aligner (BWA, v. 0.7.17; Li and Durbin, 2009). The duplication reads of BWA output files were marked using the markdup module from SAMtools (v. 1.11; Li et al., 2009). The Binary Alignment Map (BAM) files were used as the input of the Genome Analysis Toolkit indel realigner (v. 3.8) to perform local realignment around indel regions (McKenna et al., 2010; DePristo et al., 2011). Finally, each sample obtained from both sequencers was subsampled to the same total reads (mapped reads+unmapped reads) for subsequent mapDamage analysis (Ginolhac et al., 2011; Jonsson et al., 2013).

#### DNA Damage Patterns

The mapDamage (v. 2.0.6) program was processed to estimate the DNA damage pattern and rescale the quality scores of likely damaged positions in reads (Jonsson et al., 2013). Four key damage parameters, θ, δS, δD, and λ, were estimated using the Bayesian method. θ estimates the mean difference rate between the reference and the sample not caused by DNA damage. δS and δD estimate the cytosine deamination probability in single- and double-strand contexts, respectively. λ estimates the probability of terminating an overhang. These parameters were then used for bases recalibration, and the obtained BAM files were used for downstream analysis.

#### Read Duplication, Endogenous DNA Content, and Error Rate

We used the markdup module from the SAMtools program to mark the duplication reads arising from the PCR amplification process (Bonfield et al., 2021; Danecek et al., 2021). Then, the error rate, duplicate reads, and read count that mapped and unmapped to the human genome were calculated using the stats module from SAMtools. The duplication rate is defined as the ratio of the number of duplicate reads and reads mapped to the human genome. The endogenous rate is defined as the ratio of the number of reads mapped to the human genome and total reads. The error rate is defined as the ratio of mismatch bases and bases that match the human reference genome.

#### Library Complexity

The library complexity was defined as the number of distinct reads that can be observed in a given set of sequenced reads. We used the lc_extrap module from the preseq program to estimate the library complexity that implements a nonparametric empirical Bayes estimator to predict the complexity of sequencing libraries from very shallow sequencing runs (Daley and Smith, 2013). All of the mapped reads were used for predicting the libraries’ complexity.

#### Sequencing Bias

Two methods were used to study whether the two sequencing platforms are biased toward specific sequences. K-mer was used to indicate the characteristics of a library. We compared the 6-mer frequencies of the same samples between two sequencing platforms. Specifically, 100,000 reads were randomly sampled for each sample for 6-mer analysis using SAMtools, seqtk (v. 1.3) and Jellyfish (v. 2.3.0; Li et al., 2009; Marcais and Kingsford, 2011; Li, 2017). Besides this, we compared the sequencing depth and coverage of the same samples between two platforms using BEDtools (v. 2.30.0; Quinlan and Hall, 2010; Quinlan, 2014). Specifically, the reference genome was divided into 100-kb windows, and then sequencing depth and coverage were calculated in each window for each sample.

#### Population Genetic Analysis

We clipped four bases from both ends of each read from rescaled BAM files to avoid an excess of remaining C->T and G->A transitions at the ends of the sequences using trimBam implemented in BamUtil (v. 1.0.14; Jun et al., 2015). Then, we generated pseudo-haploid calls for each sample by using parameter—RandomHaploid in pileupCaller software.^1^ For population genetic analyses, we leveraged principal component analysis (PCA) and *f*-statistics analysis. For the overall population structure, we carried out the smartpca from EIGENSOFT (v. 16,000) using default parameters and lsqproject: YES (Patterson et al., 2006). To further quantify the differences in genetic relationship, we used the *qpDstat* implemented in ADMIXTOOLS (v. 900) in the form of *f_4_*(Mbuti, X, MGISEQ-2000, X-Ten) using default parameters and *f_4_*-mode: YES (Patterson et al., 2012).

## Result and Discussion

The quality of raw sequencing data was assessed using FastQC (v. 0.11.5; Andrews, 2010). The sequence quality of both platforms was similar and acceptable although X-Ten showed a higher base quality than MGISEQ-2000. The average percentage of over Q20 and over Q30 for MGISEQ-2000 were 97.73 and 87.44%. The average percentage of over Q20 and over Q30 for X-Ten were 99.29 and 92.06%. The sequencing depths of the MGISEQ-2000 platform for the samples EA1102, EA1104, EA1107, and F90914 are 0.046, 0.040, 0.016, and 0.087, respectively. The sequencing depths of the X-Ten platform for these samples are 0.043, 0.035, 0.016, and 0.078, respectively.

The same samples from both platforms were subsampled to the same total reads, and several key statistics were calculated using the stats module from SAMtools and mapDamage (Li et al., 2009; Jonsson et al., 2013). We found no significant difference in duplication rate and λ between the two platforms (Table 2). Significant but slight differences were observed in the unique endogenous rate (slightly higher for MGISEQ-2000), θ, δS, and δD between platforms. Significant differences were observed in average base quality and error rate (higher base quality and lower error rate for X-Ten). Although we observed slight differences between the two platforms on θ, δS, and δD, it is not clear which platform is closer to the actual value.

**Table 2:** Summary statistics of key parameters.

Sample	Platform	Total reads	Reads mapped	Reads unmapped	Reads duplicated	Error rate	Average quality	Duplicate rate	Endogenous rate	Unique endogenous rate	Theta (θ)	DeltaD (δD)	DeltaS (δS)	Lambda (λ)
EA1102	MGISEQ-2000	3.37E+07	1.44E+06	3.23E+07	1.92E+05	6.32E−03	38.3	0.1329	0.0427	0.0371	0.0086	0.0100	0.3064	0.3701
	X-Ten	3.37E+07	1.29E+06	3.24E+07	1.95E+05	5.45E−03	39.8	0.1511	0.0383	0.0325	0.0089	0.0088	0.2948	0.3805
EA1104	MGISEQ-2000	3.38E+07	1.23E+06	3.26E+07	1.29E+05	5.96E−03	38.2	0.1051	0.0364	0.0325	0.0048	0.0085	0.3416	0.4070
	X-Ten	3.38E+07	1.11E+06	3.27E+07	1.53E+05	4.87E−03	39.8	0.1386	0.0327	0.0282	0.0048	0.0077	0.3317	0.4081
EA1107	MGISEQ-2000	4.11E+07	5.52E+05	4.05E+07	1.37E+05	5.76E−03	38.1	0.2483	0.0134	0.0101	0.0049	0.0081	0.3499	0.4335
	X-Ten	4.11E+07	4.89E+05	4.06E+07	1.09E+05	4.90E−03	39.9	0.2237	0.0119	0.0092	0.0053	0.0073	0.3215	0.4308
F90914	MGISEQ-2000	5.45E+07	2.88E+06	5.16E+07	3.71E+05	5.63E−03	39.4	0.1288	0.0529	0.0461	0.0044	0.0091	0.2973	0.3921
	X-Ten	5.45E+07	2.61E+06	5.19E+07	3.96E+05	4.62E−03	40.6	0.1519	0.0478	0.0405	0.0049	0.0080	0.2881	0.3931
Value of *p*						0.0004	0.0012	0.3984	0.0175	0.0332	0.0393	0.0017	0.0474	0.4498

**Figure 1:**
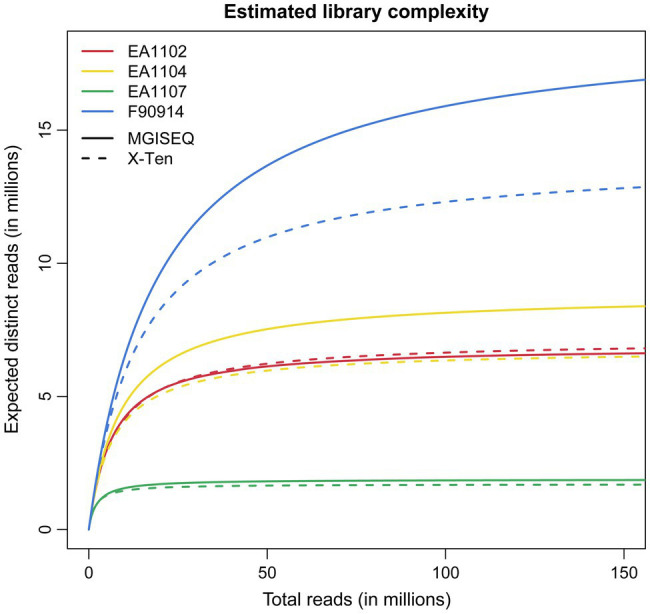
Library complexity curves described as the expected distinct reads as the function of the total reads. These curves were estimated by all of the mapped reads in BAM files using the lc_extrap module from preseq (Daley and Smith, 2013).

The library complexity of the two samples EA1102 and EA1107 is consistent between two platforms (Figure 1) although for the library complexity of the other two samples EA1104 and F90914, MGISEQ-2000 provided more libraries than X-Ten. It is noteworthy that similar results are reported in previous studies comparing the performance of BGISEQ500 and Illumina Hiseq2000 on paleogenomics (Mak et al., 2017). A previous study (Mak et al., 2017) hypothesized that the difference in complexities between the two platforms was caused by a great number of PCR cycles used for amplifying Illumina libraries (Meyer and Kircher, 2010), but we showed it was probably not the reason because we still found the difference even when we ran the same cycles for PCR amplifying in two platforms. The difference in library complexities might be due to the different sequencing strategies used by the two platforms (Porreca, 2010; Goodwin et al., 2016), which needs to be further investigated. Besides this, we found there were differences in length distribution of sequenced reads between two platforms, which indicated that there was length bias between two platforms, which may help explain the result observed (Supplementary Figure S1).

**Figure 2:**
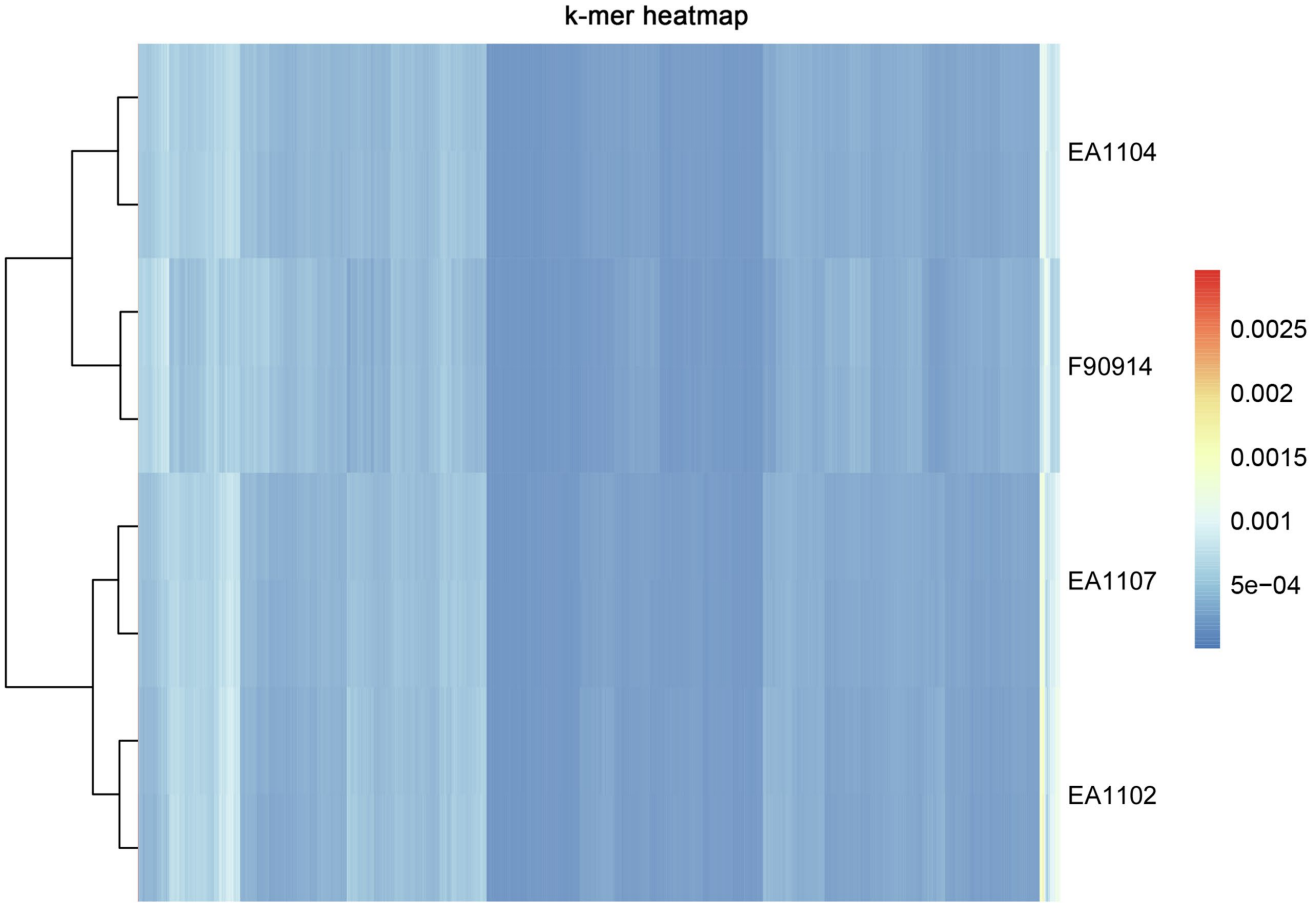
Hierarchical clustering heat map of 6-mer analysis. Libraries were clustered by the frequency of 6-mer using the pheatmap package in the R software. K-mer analysis was processed by the Jellyfish program (Marcais and Kingsford, 2011).

**Figure 3:**
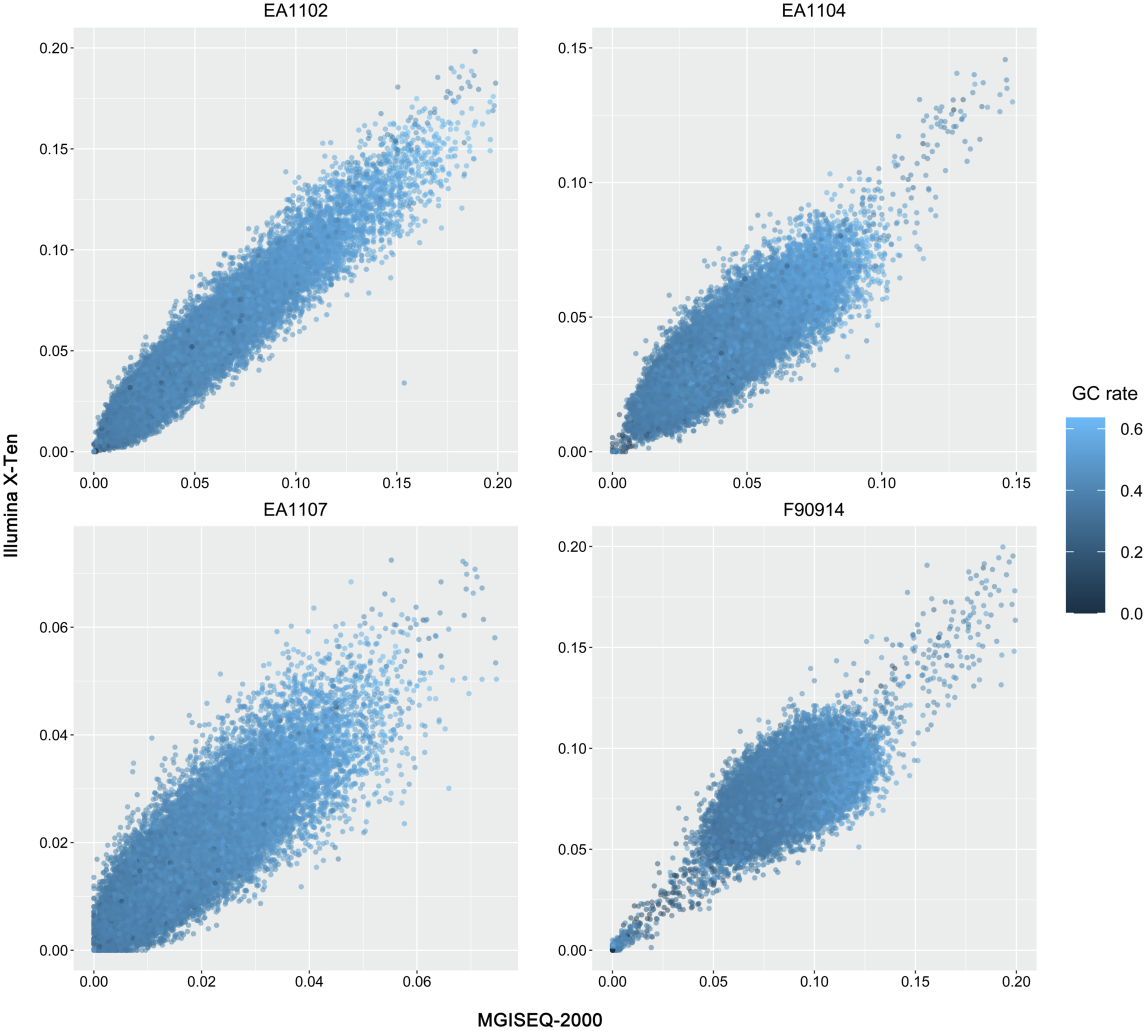
The dot plots of sequencing coverage rate of MGISEQ-2000 vs. Illumina X-Ten in 100-kb windows. The color of each dot represents the GC content in each window.

To further explore whether there are method-specific biases in sequencing different regions of the reference genome, we first processed the Jellyfish program to calculate the 6-mer frequency of each sample (Figure 2; Marcais and Kingsford, 2011). All the sample pairs were clustered together suggesting that 6-mer frequency was consistent between the two platforms. Next, we compared the sequencing depth and coverage of samples between two platforms in each 100-kb window across the whole reference genome. In all samples, we observed high consistency in sequencing depth and coverage between the two platforms (Figure 3, Supplementary Figure S2). Samples from both platforms also correlated well with the GC content of the reference genome in each window. The results together confirm that there was no significant method-specific bias between the two platforms.

**Figure 4:**
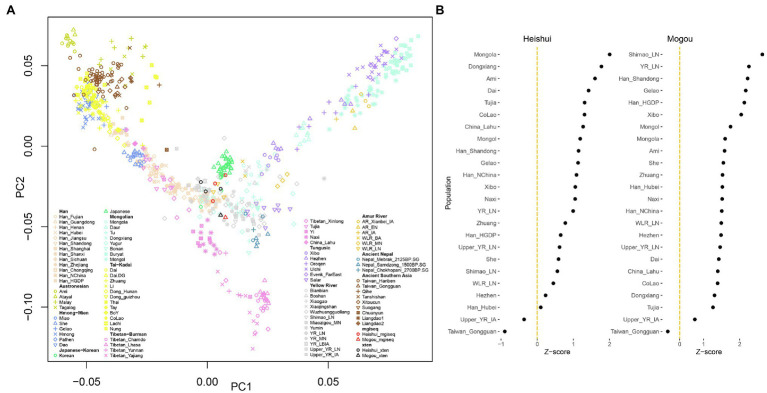
Overview of population genetic structure. **(A)** Principal component analysis plot of present-day and ancient population in East Asia. The libraries sequenced by MGISEQ-2000 and Illumina X-Ten are colored by red and black, respectively. **(B)** *f*_4_-statistics measured the relationship between MGISEQ-2000 and Illumina X-Ten with various populations in the form of *f*_4_(Mbuti, X; MGISEQ-2000, Illumina X-Ten).

Ancient DNA is widely used in studies of population genetics. To further test whether the genetic information obtained from the two platforms is consistent in the analysis of population genetics, we used the smartpca program to explore the overall population structure (Patterson et al., 2012; Wang et al., 2021). The same samples from the two platforms were generally projected closely together but not exactly at the same coordinates in the PCA plot (Figure 4A). A more refined analysis leveraged the *f_4_*-statistics in the form of *f_4_* (Mbuti, X, MGISEQ-2000, X-Ten). The Z-scores of *f_4_* statistics deviated from 0 but were smaller than |3|, indicating that there were differences between samples from two platforms, but the differences were not statistically significant (Figure 4B; Patterson et al., 2006; Peter, 2016). We hypothesized that this may be due to the low sequencing depth because we found the sample with higher sequencing depth tended to have higher correlations between platforms. As an alternative explanation, this might be caused by a slightly higher error rate in the MGISEQ-2000 platform.

In conclusion, our study evaluated the potential of using MGISEQ-2000 as an alternative sequencing platform for paleogenomics studies for the first time. We found there is no significant difference or only slight but significant differences on most of the key parameters that are crucial for paleogenomics studies. These results are consistent with previous studies comparing other BGI platforms with Illumina’s sequencing platforms (Mak et al., 2017). Our results with the previous study together indicate that the BGI series tends to provide higher library complexity and a slightly higher error rate than the Illumina series although how these two points affect downstream analysis remains unclear and requires further discussion. We observed only small differences in genetic information obtained from the two platforms in population genetics. Although we hypothesized that this might be caused by low sequencing depth, as another explanation, this may be caused by the difference in error rate between platforms. Considering that our study was only based on four samples with approximate archaeological ages, these results may not reflect all situations of ancient samples. Although the use of MGISEQ-2000 in population genetics needs further exploration, we note that MGISEQ-2000 can be used as a potential sequencer for most paleogenomics research.

## Data Availability Statement

The datasets presented in this study can be found in online repositories. The name of the repository and accession number can be found at: https://bigd.big.ac.cn/gsa, access numbers: HRA001091, HRA001090.

## Ethics Statement

The studies involving human participants were reviewed and approved by Xiamen University (Approval Number: XDYX2019009). The participants provided their written informed consent to participate in this study.

## Author Contributions

C-CW and S-QW designed this study. KZ, C-CW, and S-QW wrote the manuscript. PD, JX, XR, CS, YT, YD, YX, HM, and S-QW collected the samples. PD, JX, XR, CS, YT, YD, YX, HM, and S-QW conducted the experiment. KZ and C-CW analyzed the data. All authors reviewed the manuscript.

## Funding

The work was funded by National Key R&D Program of China (2020YFC1521607), the National Natural Science Foundation of China (32070576, 31801040), Major Project of National Social Science Foundation of China (20&ZD212), the Scientific and Technology Committee of Shanghai Municipality (18490750300), the National Key R&D Program (2020YFE0201600), Shanghai Municipal Science and Technology Major Project (2017SHZDZX01), the 111 Project (B13016), Nanqiang Outstanding Young Talents Program of Xiamen University (X2123302), the Major project of National Social Science Foundation of China (20&ZD248), a European Research Council (ERC) grant to D. Xu (ERC-2019-ADG-883700-TRAM), and Fundamental Research Funds for the Central Universities (ZK1144).

## Conflict of Interest

The authors declare that the research was conducted in the absence of any commercial or financial relationships that could be construed as a potential conflict of interest.

The reviewer JH declared a past co-authorship with the authors KZ, C-CW, KZ, and S-QW to the handling editor.

## Publisher’s Note

All claims expressed in this article are solely those of the authors and do not necessarily represent those of their affiliated organizations, or those of the publisher, the editors and the reviewers. Any product that may be evaluated in this article, or claim that may be made by its manufacturer, is not guaranteed or endorsed by the publisher.

## Footnotes

^1^ https://github.com/stschiff/sequenceTools
