# Supplementary material for: Comparative Performance of the MGISEQ-2000 and Illumina X-Ten Sequencing Platforms for Paleogenomics
Source: Front Genet. 2021 Oct 4;12:745508. doi: 10.3389/fgene.2021.745508 (PMC8521044; doi:10.3389/fgene.2021.745508)
Supplement: Supplementary file 1 [file Data_Sheet_1.docx]

# Supplementary Materials


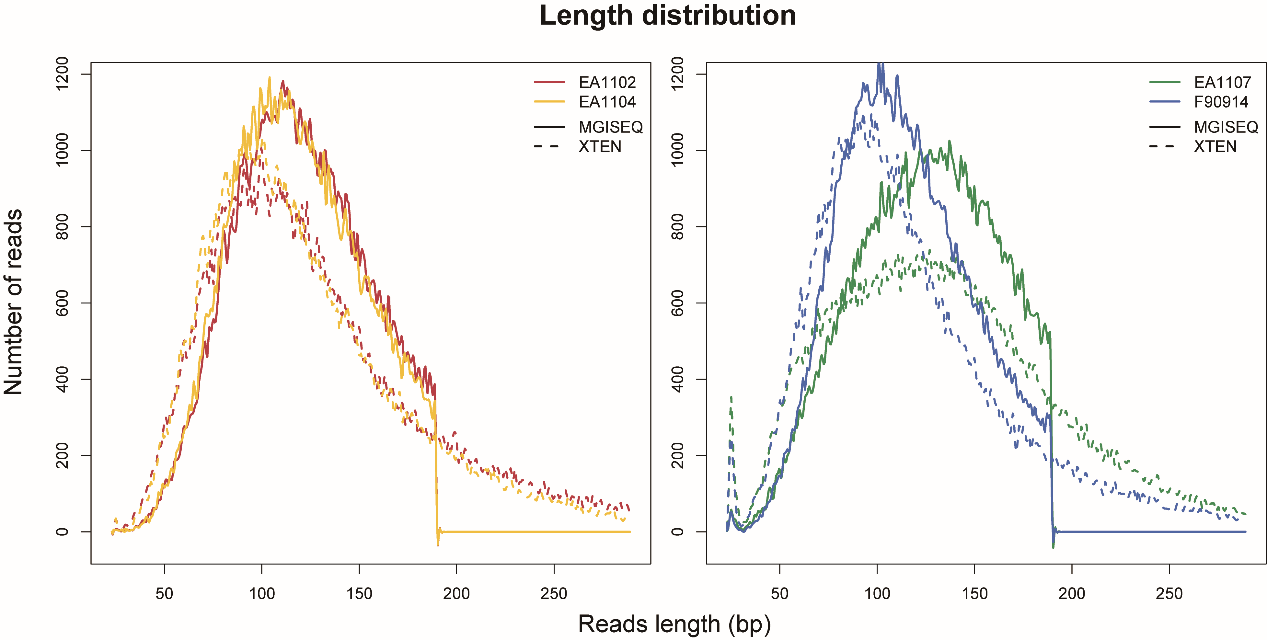


**Figure S1.** Length distribution of merged reads of four samples from two platforms.


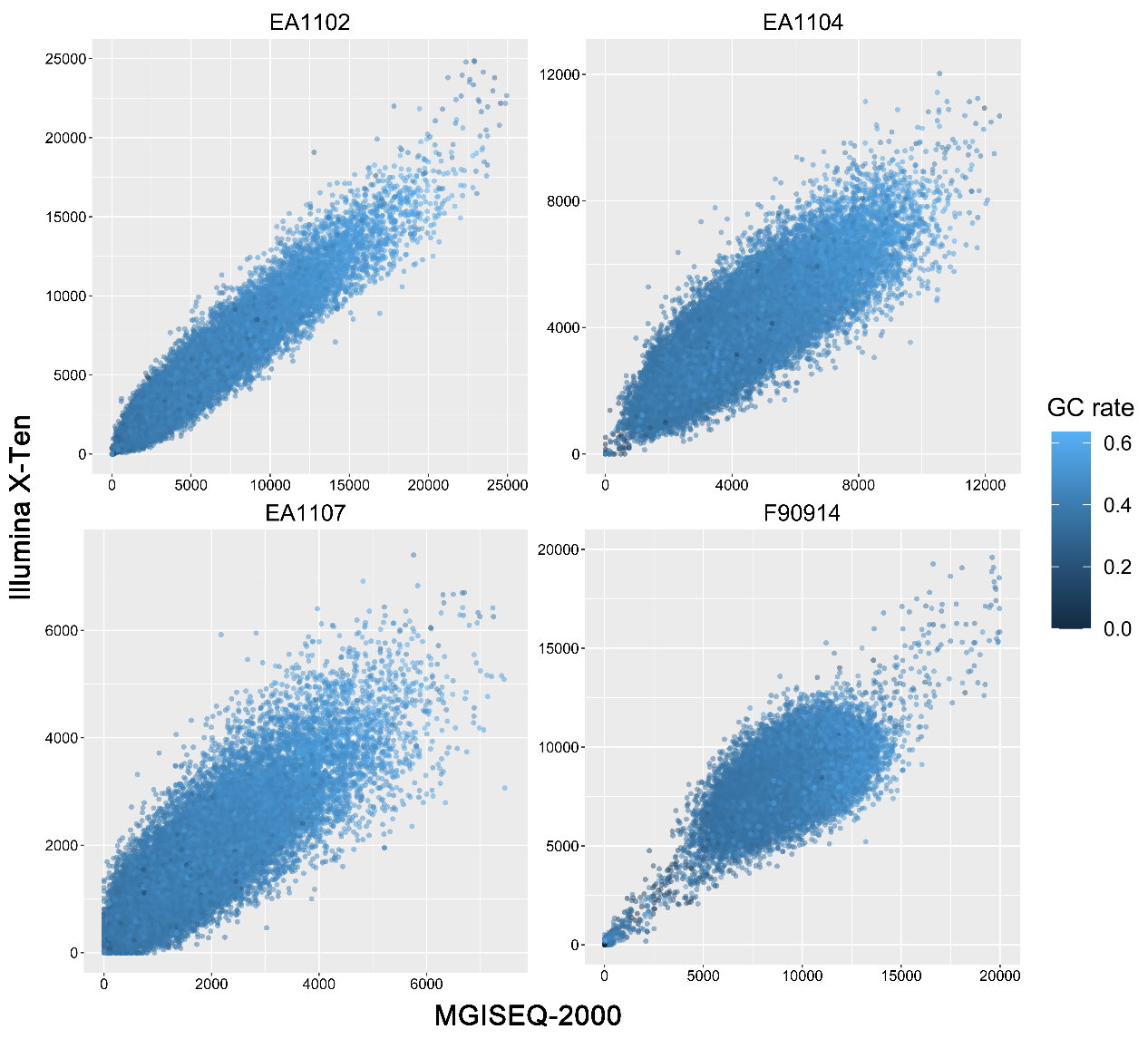


**Figure S2.** The dot plot of sequencing depth of MGISEQ-2000 vs Illumina X-Ten in 100kb windows. The color of each dot represents the GC content in each window.
